# Evaluating the Role of Magnification in Minimising Tooth Substance Loss During Endodontic Access Cavity Preparation: An In Vitro Volumetric Analysis Using CBCT and IOS

**DOI:** 10.3390/medicina62081594

**Published:** 2026-08-19

**Authors:** Rafaela Martins, Abayomi Omokeji Baruwa, Karla Baumotte, António Ginjeira

**Affiliations:** 1 Faculdade de Medicina Dentária, Universidade de Lisboa, 1600-277 Lisboa, Portugalaginjeira@edu.ulisboa.pt (A.G.); 2 Department of Clinical Sciences, College of Dentistry, Ajman University, Ajman 346, United Arab Emirates

**Keywords:** endodontics, cone beam computed tomography, access cavity, intraoral scanner, mandibular incisors, operating microscope

## Abstract

*Background and Objectives*: The aim of this study was to evaluate the volume of dental tissue removed during conventional versus microscope-assisted coronal access cavity preparation by an undergraduate student in extracted mandibular incisors, and to compare the volumetric assessment capabilities of cone beam computed tomography (CBCT) and intraoral scanner (IOS) for this application. *Materials and Methods*: Thirty extracted mandibular incisors were randomly allocated to two equal groups: Group 1 (G1), access cavity preparation without magnification; and Group 2 (G2), access cavity preparation with the aid of an operating microscope. Volumetric analysis was performed using CBCT (RAYSCAN^®^ Alpha Plus 130) and an intraoral scanner (3Shape TRIOS^®^ 3) before and after access cavity preparation. Volume loss was calculated for each group. Differences in groups were assessed using the independent-samples Student’s *t*-test for CBCT data and the Mann–Whitney U test for IOS data. Reproducibility between methods was evaluated using the intraclass correlation coefficient (ICC). *Results*: The CBCT analysis revealed significantly greater tissue loss in G1 (mean 14.93 mm^3^, SD 4.42) than in G2 (mean 7.64 mm^3^, SD 2.72) (mean difference 7.29 mm^3^, 95% CI, 4.54–10.04; *p* < 0.001; Cohen’s d = 1.99, 95% CI, 1.10–2.88). The IOS analysis did not detect a statistically significant between-group difference (G1: mean 5.37 mm^3^ vs. G2: mean 4.55 mm^3^; *p* = 0.436). The ICC for volume loss between methods was 0.011 (95% CI: −0.150 to 0.237), below the threshold conventionally used to indicate acceptable reliability; because CBCT and IOS did not sample identical anatomical extents, this should be interpreted as limited agreement specific to the present protocols rather than general evidence of poor concordance between the two imaging methods. *Conclusions*: Within the limitations of this exploratory, single-operator in vitro study, microscope-assisted access cavity preparation resulted in significantly less CBCT-measured volumetric tissue loss than conventional access in mandibular incisors.

## 1. Introduction

Access cavity preparation is the first and most critical operative stage of root canal treatment. Its primary objective is to provide unobstructed access to the pulp chamber and root canal orifices, enabling their identification, debridement, and preparation. Achieving an adequate access cavity directly determines the feasibility of subsequent chemomechanical preparation, the quality of irrigant delivery, and the long-term structural integrity of the treated tooth [1,2]. A restrictive or improperly designed access cavity compromises canal location, increases the mechanical fatigue of nickel–titanium instruments, and hinders the delivery of irrigants to the apical third of the root canal system [3,4].

Contemporary endodontic practice has been profoundly influenced by the concept of minimally invasive dentistry, which emphasises the preservation of sound dental tissue at every stage of treatment [5]. Preservation of dentinal structure is directly associated with resistance to tooth fracture following root canal treatment, particularly in teeth with limited residual structure such as mandibular incisors [6,7]. Accordingly, the design of access cavities should not follow a predetermined geometric form based on clinician preference; instead, it must be individualised for each tooth and each patient [1,8]. In pursuit of more conservative and precise preparations, the operating microscope has become an increasingly important adjunct in endodontic practice. Introduced into endodontic procedures during the late 1970s, the operating microscope provides superior magnification and co-axial illumination of the operative field, enabling clinicians to perform more controlled and tissue-sparing procedures [4,9]. Its utility has been demonstrated in the location of calcified or additional canals, the removal of pulp chamber obstructions, and the retrieval of fractured instruments [10].

Quantification of dental tissue loss during access cavity preparation has primarily been performed using micro-computed tomography (micro-CT), which offers high spatial resolution and accurate three-dimensional volumetric data [11]. However, micro-CT is not applicable to in vivo research owing to prohibitive radiation doses and strict specimen size limitations [5,11], whilst cone beam computed tomography (CBCT) represents a clinically applicable alternative, providing detailed images of dental hard tissues at radiation doses compatible with clinical use [12,13]. To date, limited studies have employed CBCT with a radiation dose compatible with in vivo translational study to quantify the effect of microscope magnification on volumetric tissue loss specifically in the mandibular incisors, a tooth group whose narrow mesiodistal dimension and high prevalence of a second canal make conservative access particularly challenging [14]. The intraoral scanner (IOS) represents a further technological option for acquiring three-dimensional surface models of teeth and surrounding tissues [15]. IOS, a chairside and radiation-free alternative, has demonstrated an accuracy comparable to conventional impression techniques for many applications; however, its utility for capturing deep, narrow endodontic access volumes remains unexplored [16].

To our knowledge, no previous study has combined CBCT and intraoral scanning to evaluate microscope-assisted access cavity preparation specifically in mandibular incisors. The present study therefore aimed to evaluate the volume of dental tissue removed during conventional versus microscope-assisted access cavity preparation in the coronal region of extracted mandibular incisors, using both CBCT and IOS for volumetric determination. A secondary aim was to assess if the IOS can reproduce CBCT-derived volumetric estimates.

## 2. Materials and Methods

### 2.1. Sample Selection and Preparation

**Figure 1:**
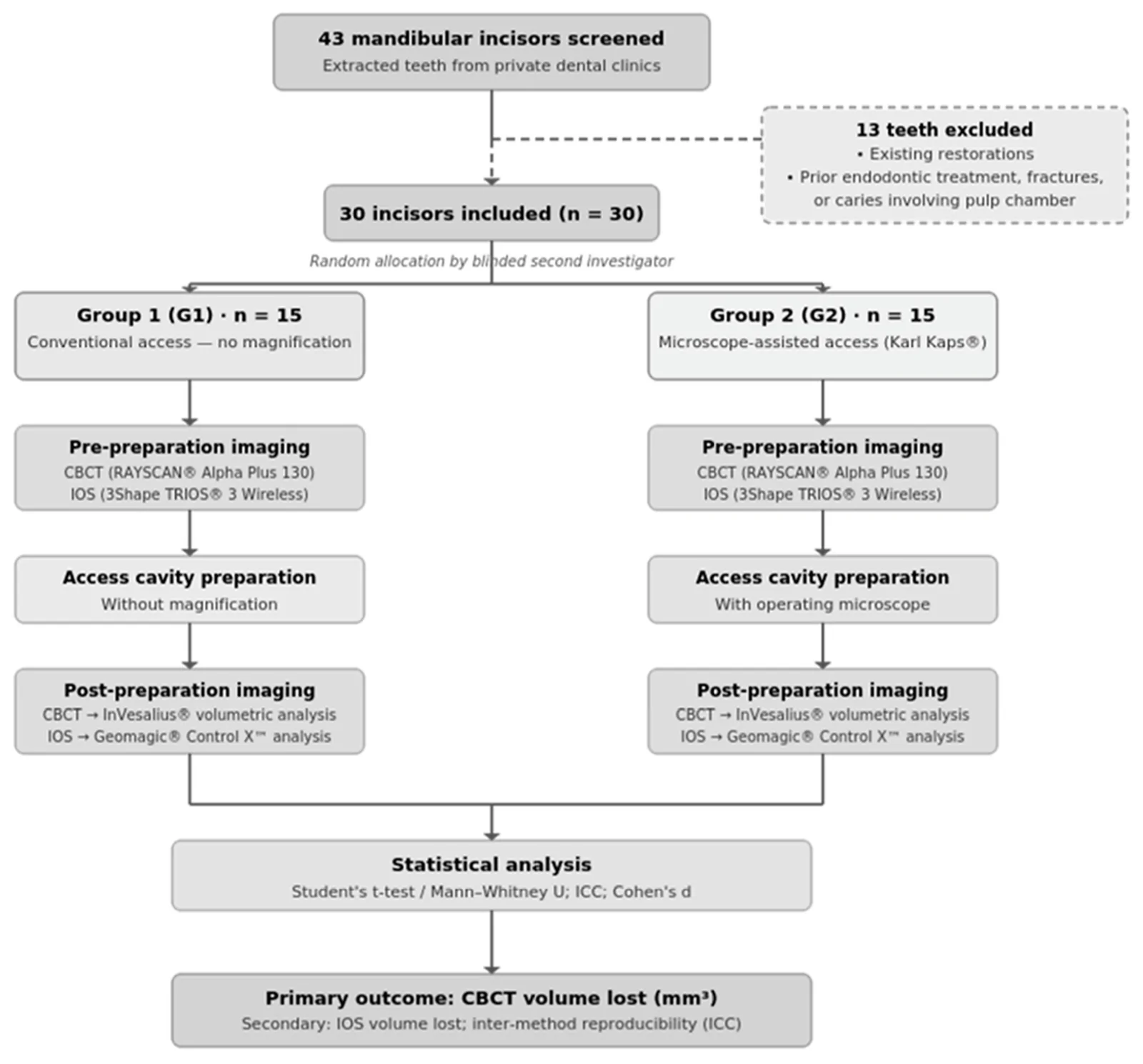
Study flow diagram.

This study was performed according to the Preferred Reporting Items for Laboratory Studies in Endodontology (PRILE) 2021 guidelines and the chart is attached as supplementary [17]. The study design and participant flow are summarised in Figure 1. Teeth originally extracted for reasons unrelated to this study were collected from private dental clinics between March 2022 and February 2023 and were fully anonymized at the point of collection with no identifying information retained. Teeth were subsequently stored in physiologic saline at 4 °C. The research ethics committee waiver was obtained from the Faculty of Dental Medicine, University of Lisbon, as anonymized specimens from the extracted-tooth bank are exempt from individual ethics committee review under institutional policy (Ref. BD-CE-FMDUL202603), as was in force at the time of the study. From a pool of anonymized extracted human teeth, forty-three mandibular incisors were screened for inclusion. Teeth presenting existing restorations, previous endodontic treatment, visible fractures, or extensive carious lesions involving the pulp chamber were excluded. Following exclusion of thirteen teeth based on these criteria, the final sample comprised thirty mandibular incisors. Mandibular central and lateral incisors were analysed as a single group, as both present a comparable mesiodistal crown dimension and root canal configuration. All specimens were cleaned by ultrasonic scaling, stored in individual numbered containers with distilled water, and periapical radiographs were obtained for documentation. As this study was designed as an exploratory in vitro investigation, a formal a priori power calculation was not performed because, at the time of protocol design, no comparable published data existed on CBCT-quantified volumetric substance loss during magnification-assisted access cavity preparation in mandibular incisors specifically; the closest available estimate was from a study using a different tooth model and outcome metric [18]. The sample size was therefore set pragmatically and should be regarded as providing preliminary effect estimates.

### 2.2. Image Acquisition Using CBCT and Intraoral Scanning

CBCT and IOS images were acquired at two time points for each specimen: before and after access cavity preparation. All specimens were placed in individual acrylic containers numbered 1 to 30, and each tooth was embedded in alginate to create a stabilising support that standardised specimen positioning and ensured reproducible image acquisition across both scanning sessions.

CBCT imaging was performed using a RAYSCAN^®^ Alpha Plus 130 unit (Ray America PC CBCT, Hwaseong-si, Republic of Korea) with the following acquisition parameters: voxel size 100 μm, field of view 4 × 5 cm, exposure time 14 s, tube voltage 85 kVp, and tube current 10 mA.

Three-dimensional surface images were additionally acquired using a 3Shape TRIOS^®^ 3 Wireless intraoral scanner (Straumann Group Digital Solutions, Copenhagen, Denmark), operated in conjunction with a DELL^®^ Precision 7560 laptop (Dell, Round Rock, TX, USA, Intel Core i7-11850 processor, Intel, Santa Clara, CA, USA). Each specimen was secured in a prefabricated silicone support in a standardised vertical position prior to scanning to ensure reproducible surface acquisition. IOS images were exported as STL files for subsequent volumetric analysis.

### 2.3. Access Cavity Preparation

Simple random allocation into two groups was performed by K.B., who had no knowledge of specimen anatomical characteristics. Specimens were prepared in randomised order and the operator received prior instructions and training on access cavity preparation to the study. Each diamond bur was replaced after a maximum of six teeth, alternating between groups to avoid confounding bur wear with preparation technique. In Group 1 (G1), access cavities were prepared without magnification. In Group 2 (G2), access cavities were prepared with the aid of an operating microscope at a magnification of ×10 (Karl Kaps^®^, GmbH and Co. KG, Asslar, Germany). A custom silicone support was fabricated to standardise tooth positioning throughout the access cavity preparation stage.

Access cavities were prepared using size 10 spherical diamond burs (M 801.314.010) and size 10 conical diamond burs (Z12 M858.314.010) (R and S^®^, Toulouse, France) at high speed with water coolant. Each bur was used for a maximum of six teeth. Access cavity preparation was initiated by identifying the entry point located 2 to 3 mm above the cingulum. A spherical diamond bur was first applied with a drilling direction perpendicular to the long axis of the tooth, which was then progressively adjusted to become nearly parallel until the pulp chamber was entered. The complete roof of the pulp chamber was removed, and conical diamond burs were subsequently used to refine the final triangular outline form and to regularise the axial walls leading to the root canal orifices. Successful access to the root canal system was confirmed using size 10 stainless-steel manual K-files (THOMAS^®^, FFDM Pneumat, Bourges, France, 25 mm, ISO 010).

All access cavity preparations were performed by a single undergraduate dental student under the supervision of a specialist endodontist. A single operator was chosen to eliminate inter-operator variability as a confounder in the volumetric comparisons; an undergraduate student was selected because the operator profile is representative of the training stage at which access cavity technique, including microscope use, is first acquired. This choice therefore maximises internal validity for the technique comparison over external validity to experienced clinician.

### 2.4. Analysis of Lost Dental Tissue Volume

Assessment of dental tissue loss from CBCT imaging was performed using Digital Imaging and Communications in Medicine (DICOM)-format datasets imported into InVesalius^®^ software (InVesalius 3.1, Centro de Tecnologia da Informação Renato Archer, Campinas, Brazil) on a Windows operating system [14]. The volumes of the pulp chamber and the acrylic container with alginate support were excluded from volumetric calculations by applying a density threshold filter that retained only voxels with radiographic density values corresponding to enamel, dentine, and cementum. As mineral density varied across specimens, threshold settings were adjusted individually for each tooth, with minimum values in the range of 70 to 105 and maximum values in the range of 245 to 255 manufacturer-native greyscale units. These values were refined iteratively until the alginate support was excluded entirely from the reconstructed image and only dental hard tissue components were represented. This specimen-specific threshold adjustment introduces a degree of operator-dependent variability; reproducibility of the segmentation step, either between examiners or on repeated measurement by the same operator, was not formally assessed, and this is acknowledged as a limitation. Three-dimensional surface reconstructions were then generated using the region-of-interest selection and surface creation tools within the software. Formal calibration to Hounsfield units using a density phantom was not performed, which limits comparability of the reported threshold ranges with studies using calibrated Hounsfield-unit thresholds. Volumetric outputs from InVesalius^®^ were recorded at a 1000× scale relative to the reported unit and were divided by 1000 during data export to obtain the volume in mm^3^ reported throughout.

The initial volume of enamel, dentine, and cementum (V*i*), expressed in cubic millimetres, was recorded for each tooth from the pre-preparation CBCT dataset. Following access cavity preparation, a second CBCT scan was performed and analysed using the same previously established threshold settings to obtain the final volume (V*f*). The volume of dental tissue lost as determined by CBCT analysis (V*p*) was calculated as follows: V*p* = V*i* − V*f*.

For intraoral scanner analysis, Standard Tessellation Language (STL) files were imported into Geomagic^®^ Control X™ software (Version 2020.1.1, 3D Systems Inc., Rock Hill, SC, USA). The silicone support was digitally removed from each scan using the cutting tool, and the initial (V*I*) and final (V*F*) volumes were determined for each specimen. Pre- and post-preparation images were aligned using the software alignment tool (align between scan data) to ensure an identical apical reference level was applied to both scans for each specimen. The volume of dental tissue lost as determined by IOS analysis (V*P*) was calculated as V*P* = V*I* − V*F*. IOS measurements were limited to the crown and coronal root third, as the apical portion was embedded in the silicone support; accordingly, absolute IOS volumetric values are not directly comparable with CBCT measurements, which encompassed the entire tooth structure.

The operator responsible for CBCT segmentation and the operator of the IOS volumetric analysis software were aware of the group allocation because the tooth numbering was not separated from group identity during image processing.

### 2.5. Statistical Analysis

Data were entered into Microsoft Excel (Microsoft Office 365, Redmond, WA, USA) and statistical analysis was performed using SPSS software (Version 29, IBM Corporation, Armonk, NY, USA). The significance level was set at α = 0.05 throughout. Descriptive statistics included mean, standard deviation, minimum, maximum, median, and interquartile range for initial, final, and lost volumes, reported for the overall sample and stratified by access cavity preparation method and volumetric assessment technique. Graphical representations were produced using simple and grouped boxplots.

Normality of volume distribution was assessed using the Shapiro–Wilk test for each group and outcome variable. CBCT volumetric data satisfied the assumption of normality for both groups (*p* > 0.05); between-group comparisons therefore employed the independent-samples Student’s *t*-test, and paired comparisons of initial versus final volumes within each group employed the paired-samples Student’s *t*-test. IOS volumetric data deviated significantly from normality in at least one group (*p* < 0.05); between-group comparisons therefore employed the Mann–Whitney U test, and paired comparisons employed the Wilcoxon signed-rank test. Effect size for the primary CBCT between-group comparison of volume lost was calculated as Cohen’s d. Reproducibility between CBCT and IOS methods was evaluated using the intraclass correlation coefficient (ICC) with a 95% confidence interval, using a two-way mixed-effects model with absolute agreement [19]. Three post hoc sensitivity analyses were subsequently performed for the CBCT-derived volume-lost outcome: (i) tissue loss was re-expressed as a percentage of each specimen’s initial volume (volume lost/initial volume × 100) and compared between groups using the independent-samples *t*-test (CBCT) or Mann–Whitney U test (IOS), matching the test used for the corresponding absolute volumes; (ii) an analysis of covariance (ANCOVA) was performed with volume lost as the dependent variable, group as the fixed factor, and initial volume as a covariate, to assess whether baseline anatomical size confounded the group effect; and (iii) the primary between-group comparison was repeated after exclusion of the single outlying specimen.

## 3. Results

### 3.1. Volumetric Assessment Using Cone Beam Computed Tomography

**Figure 2:**
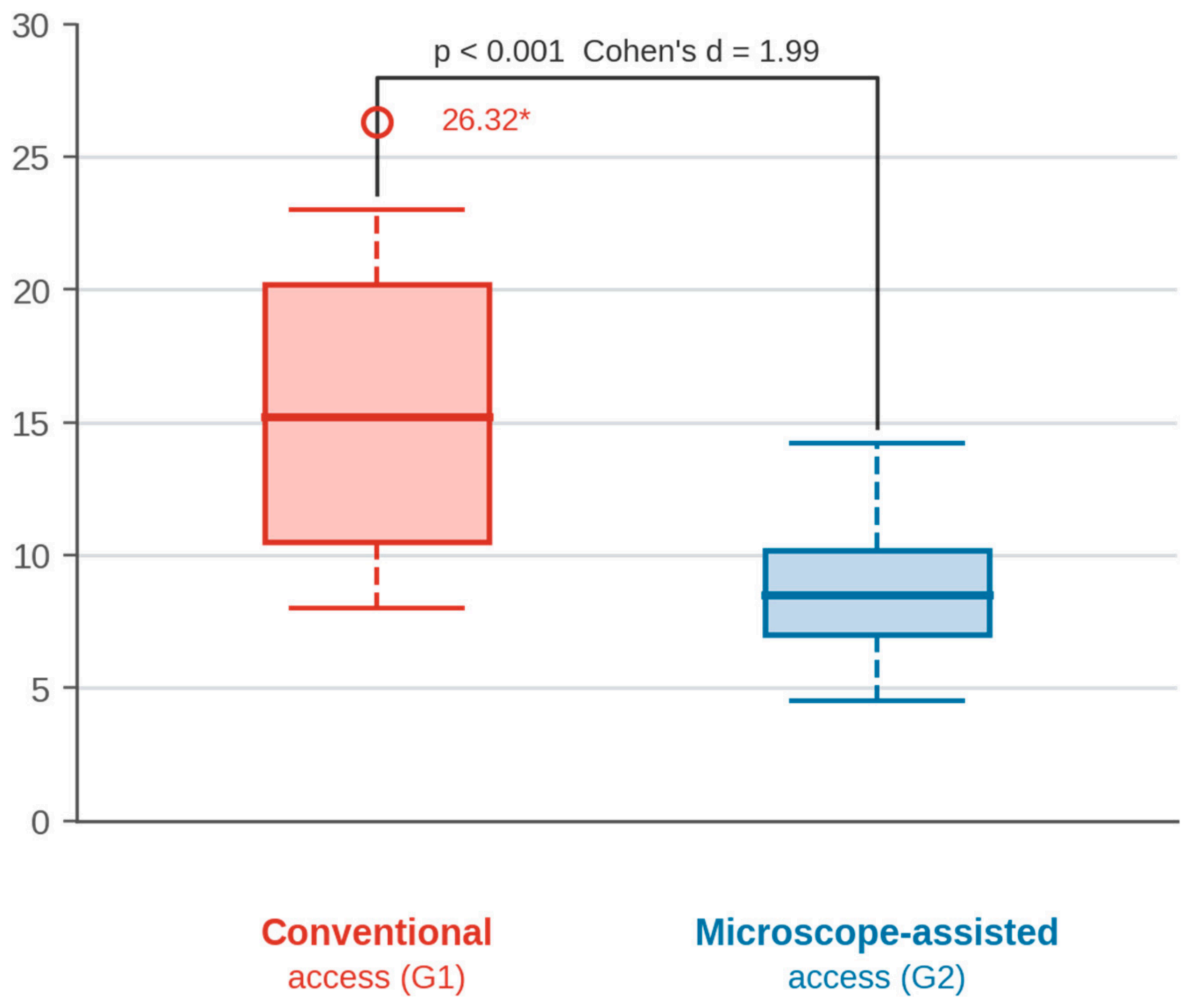
Boxplot of tissue volume lost (mm^3^) during coronal access cavity preparation as measured by cone beam computed tomography. The * represents the outlier sample.

The CBCT-derived initial and final volumes did not differ significantly between G1 and G2 (*p* = 0.197; mean difference 19.35 mm^3^, 95% CI, −10.61 to 49.31), nor did final volumes prior to within-group tissue loss being accounted for (*p* = 0.420; mean difference 12.06 mm^3^, 95% CI, −18.15 to 42.27; Table 1). The absence of a statistically significant baseline difference is consistent with, but does not by itself prove, adequate randomisations; with 15 specimens per group, the study had limited power to detect baseline imbalances. Within each group, a statistically significant reduction in volume from initial to final measurement was confirmed, reflecting successful tissue loss (*p* < 0.001 for both groups). The volume of dental tissue lost during access cavity preparation was significantly greater in G1 (conventional access; mean 14.93 mm^3^, SD 4.42, range 7.80 to 26.32 mm^3^) than in G2 (microscope-assisted access; mean 7.64 mm^3^, SD 2.72, range 4.27 to 11.93 mm^3^; mean difference 7.29 mm^3^, 95% CI, 4.54–10.04; *p* < 0.001; Cohen’s d = 1.99, 95% CI, 1.10–2.88; Table 1). The large effect size indicates a statistically significant and quantitatively large difference between preparation techniques. Substantially greater variability in tissue loss was observed in G1 compared with G2, as illustrated in Figure 2. An outlier in G1, corresponding to one specimen with a volume loss of 26.32 mm^3^, may reflect greater procedural variability in the conventional group; no specific procedural deviation was recorded for this specimen, so a specific causal mechanism cannot be confirmed.

### 3.2. Volumetric Assessment Using Intraoral Scanning

**Figure 3:**
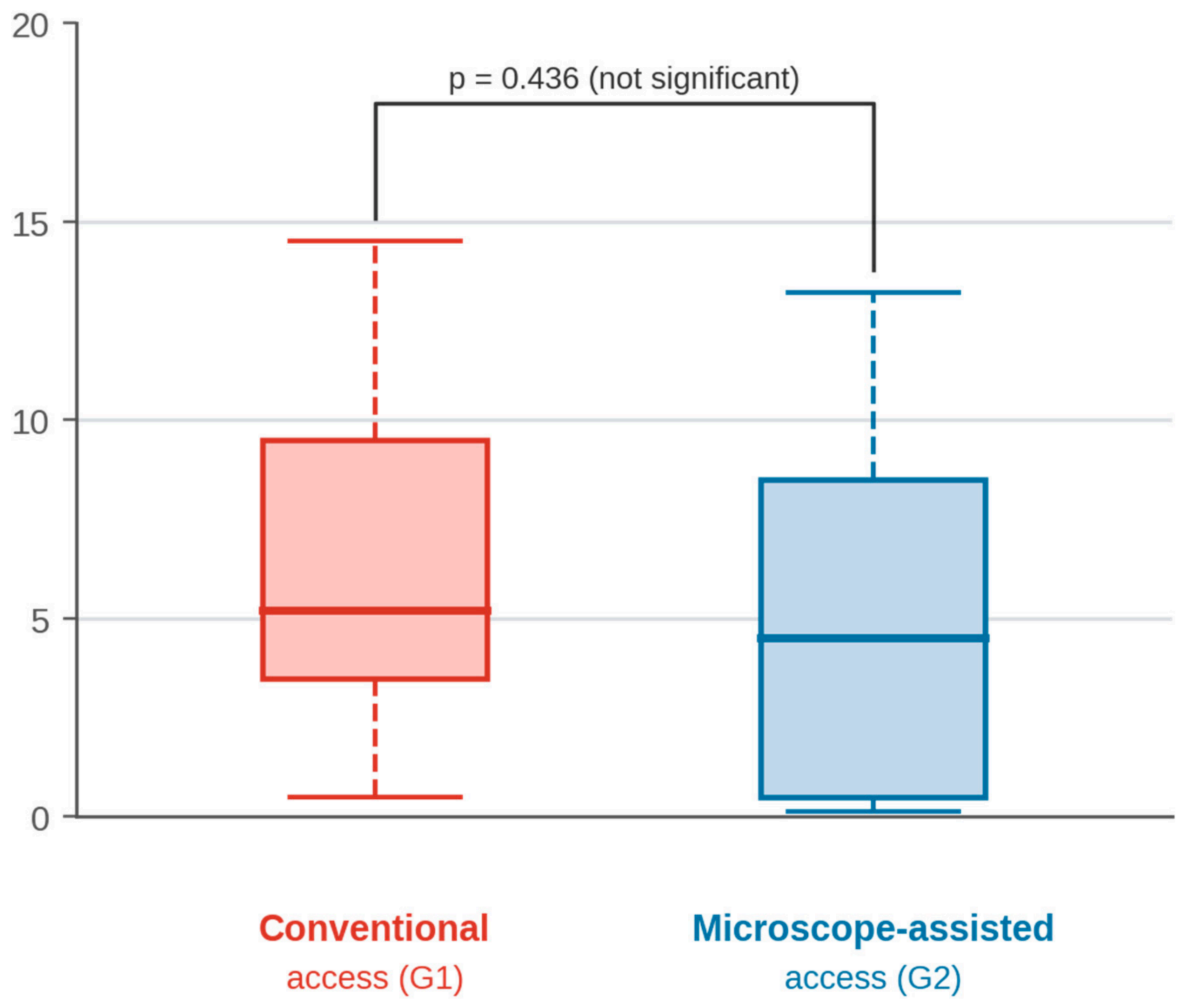
Tissue volume lost (mm^3^) during coronal access cavity preparation as measured by intraoral scanner.

The IOS-derived initial and final volumes showed no statistically significant between-group difference (Table 2; *p* = 0.838 and *p* = 0.683 for initial and final volumes, respectively), confirming baseline group comparability for this method. Within-group comparisons of initial and final IOS volumes were statistically significant in both groups (*p* < 0.001), confirming that tissue loss was detectable at the individual specimen level. The between-group comparison of IOS-derived volume lost did not reach statistical significance (G1: mean 5.37 mm^3^, SD 4.17, range 1.17 to 14.61 mm^3^ vs. G2: mean 4.55 mm^3^, SD 4.08, range 0.86 to 12.69 mm^3^; *p* = 0.436; Table 2). The distribution of volume loss values was similar between groups, as illustrated in Figure 3. These findings contrast markedly with the CBCT results: IOS did not detect a statistically significant between-group difference in tissue loss where CBCT did identify one.

### 3.3. Comparison Between Methods and Reproducibility Analysis

**Figure 4:**
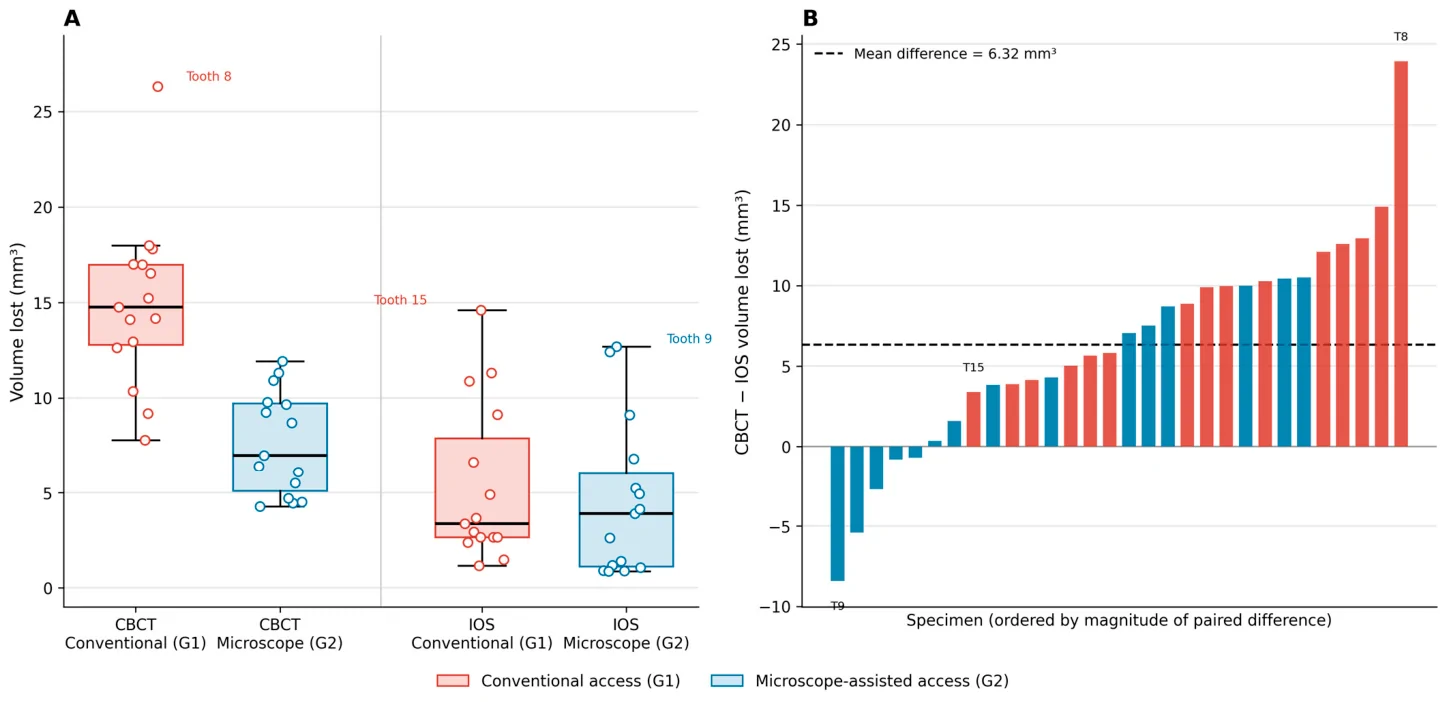
Individual-specimen volume lost (mm^3^) and paired inter-method differences. (**A**) Box-and-whisker plots (median, interquartile range) overlaid with individual observations for all 30 specimens, shown by imaging method (CBCT, IOS) and access type (G1, conventional; G2, microscope-assisted). (**B**) Paired CBCT-minus-IOS difference in volume lost per specimen, ordered by magnitude and colour-coded by group; dashed line indicates the mean paired difference.

Comparison of absolute volumetric values between CBCT and IOS revealed statistically significant differences for initial volume, final volume, and volume lost (all *p* < 0.001), consistent with the two methods sampling different anatomical extents. Figure 4 presents the individual specimen-level volume lost observations for all four measurements, enabling a direct visual comparison between methods and groups.

CBCT consistently yielded higher absolute values for all three parameters, reflecting the difference in scanning scope: CBCT measurements encompassed the entire tooth, whereas IOS measurements were limited to the crown and coronal root third. Because initial and final volumes were not measured over the same anatomical extent by the two methods, the corresponding ICC values (initial volume: ICC = 0.732, 95% CI, −0.034 to 0.934; final volume: ICC = 0.798, 95% CI, −0.047 to 0.951) do not represent agreement between equivalent measurements and are reported descriptively only; both estimates also carry wide confidence intervals spanning negative values, indicating imprecise estimation. The volume-lost ICC (0.011, 95% CI, −0.150 to 0.237; Table 3) is less affected by this anatomical mismatch, since the tissue removed during access preparation lies within the region captured by both methods; according to established criteria, ICC values below 0.50 indicate poor reliability [19]. However, the very wide confidence interval, which spans negative values, indicates that the present sample was insufficient to estimate inter-method agreement with precision, and this result should be interpreted as specific to the acquisition and analysis protocols used here rather than as a general finding of poor concordance between CBCT and IOS.

### 3.4. Sensitivity Analysis

To account for possible baseline anatomical variability between groups, three additional analyses were performed for the CBCT-derived volume-lost outcome. First, when tissue loss was normalised as a percentage of each specimen’s initial volume, the between-group difference persisted: G1 lost 6.17% ± 2.20% of the initial volume versus 3.36% ± 1.37% in G2 (independent-samples *t*-test, *p* < 0.001, Cohen’s d = 1.53; Mann–Whitney U test performed as an additional robustness check, *p* < 0.001). For IOS, the normalised difference remained non-significant (2.62% ± 2.11% in G1 versus 2.31% ± 1.96% in G2; Mann–Whitney U test, *p* = 0.534), consistent with the absolute-volume analysis. Second, an ANCOVA with initial volume as a covariate confirmed that the CBCT group effect was essentially unchanged after adjustment (adjusted mean difference 7.37 mm^3^ versus 7.29 mm^3^ unadjusted; *p* < 0.001), while initial volume itself was not a significant covariate (*p* = 0.81), indicating that baseline tooth size did not confound the primary finding. Third, the CBCT between-group difference in volume lost remained significant after exclusion of the single outlying specimen (tooth 8; mean difference 6.48 mm^3^, *p* < 0.001), confirming that this outlier was not driving the overall result.

## 4. Discussion

Mandibular incisors represent one of the least studied tooth groups for endodontic access cavity volumetric substance loss, despite their anatomical complexity. Up to 45% of these teeth have a second root canal [1,20,21]. Where two orifices are present, an intervening dentinal projection often obscures the lingual canal during conventional access preparation, prompting the recommendation to extend the preparation towards the cingulum or an alternative buccal or incisal approach [8,22]. The present study demonstrates that microscope-assisted access cavity preparation resulted in significantly less dental tissue loss than conventional preparation, corresponding to a mean reduction of approximately 49% relative to the conventional access group as quantified by CBCT volumetric analysis. This substantial effect extends the established principle that magnification promotes operative precision and tooth conservation during access cavity preparation in mandibular incisors.

The operating microscope enhances the quality of the operative field through superior magnification and co-axial illumination, enabling the clinician to distinguish dentine from pulp tissue more accurately, to minimise inadvertent removal of structurally important dentinal walls, and to locate the pulp chamber and canal orifices with greater precision [7,10]. The significantly reduced tissue loss observed in G2 is consistent with these previously mentioned benefits and is directionally consistent with findings in other tooth groups [23], although the study used a different access cavity design and an experienced operator, limiting direct comparison. Nica et al. [24] reported a mean tissue loss of approximately 10 mm^3^ in mandibular incisors using microscope-assisted access; however, that study used IOS as the sole volumetric method, a different bur protocol, and did not report operator experience level, so this figure should be compared with caution against the present IOS-derived G2 mean (4.55 mm^3^) rather than being considered a direct comparison. It is worth noting that, whilst the benefits of the operating microscope for access preparation are well supported, the primary challenge in clinical adoption relates not to the cost of the equipment but to the considerable adaptation period required for students in training or early-career clinicians to develop the ergonomic and visual skills necessary for effective use [25]. This observation has direct relevance to the present study, in which the operator was entirely inexperienced in microscope use and supports the expectation that results may differ as operator proficiency develops.

The discordance between the CBCT and IOS findings represents the most important secondary observation of this study. Whilst CBCT detected large, statistically significant differences between the groups, IOS failed to detect any significant difference. This could be due to the fact that the IOS optical acquisition system is optimised for imaging external dental surfaces and may have difficulty capturing the full geometry of deep, narrow cavities such as endodontic access preparations, particularly in small teeth such as mandibular incisors. Pinto et al. [26] reported that in deep channels, there may be regions not reached by the IOS light beam, producing incomplete spatial acquisition and therefore inaccurate volumetric determination. In addition, IOS measurements were limited to the crown and coronal third of the root, owing to partial specimen embedding, introducing variability in the scanned surface area across specimens and likely reducing statistical power. The ICC for volume loss between CBCT and IOS was 0.011, below the threshold conventionally used to indicate acceptable reliability. Part of this low value may reflect the non-equivalent measurement domains of the two methods, CBCT capturing the entire tooth versus IOS capturing only the crown and coronal root third, rather than a fundamental disagreement between the imaging methods. Importantly, all three ICC estimates were accompanied by wide confidence intervals spanning negative values, indicating that the sample was insufficient to estimate inter-method agreement with acceptable precision for any parameter. A further explanation for the non-significant IOS between-group comparison could be attributed to the smaller effect size detectable by IOS, and a smaller sample per group has limited power to detect an effect of that size with the observed variance. The absence of a significant IOS result should therefore not be attributed solely to the methodological insensitivity of IOS; inadequate statistical power is an equally plausible, non-exclusive explanation. These findings underscore the need for a dedicated reproducibility investigation with appropriate a priori power calculation. Collectively, these findings suggest that IOS, as applied in this study, was unable to detect the inter-group difference identified by CBCT for the volume-loss parameter. Whether this reflects the methodological insensitivity of IOS for deep narrow cavities, the restricted anatomical scope of IOS measurements relative to CBCT, or insufficient statistical power in the present sample remains to be determined. An alternative interpretation is that the inter-group difference in tissue loss was genuinely smaller at the crown and coronal root surface, which is only the region captured by IOS, rather than at the full-tooth level, captured by CBCT, owing to the anatomical location of access preparation. Both explanations should be considered in future study with appropriate sample sizes and standardised scanning scope.

The primary outcome of this study should be interpreted with caution as all access cavity preparations were performed by a single operator who was an undergraduate dental student with limited experience in the use of an operating microscope. Although the findings may be applicable and generalizable to individuals with a similar level of experience, they may not necessarily extend to clinicians with differing expertise levels. Within the limitations of this study, we propose as a hypothesis, rather than a conclusion supported by the present data, that the observed reduction in tissue loss in G2 may partly reflect the slower, more deliberate operative approach of a novice user learning to work with unfamiliar equipment, rather than the full potential benefit of an expert microscope-assisted technique. This would imply that an experienced microscope user could demonstrate an even greater advantage over conventional access techniques, but this remains speculative and was not tested in the present study.

Several additional limitations merit consideration such as there being no formal assessment of intra-examiner reproducibility of the segmentation procedure performed, and this is acknowledged as a limitation because threshold selection required subjective judgement per specimen; hence, a proportion of the observed CBCT volume-loss estimates may reflect measurement variability rather than true tissue loss. The absence of a formal a priori power calculation means the study may have been underpowered, particularly for the IOS analysis. Also, the specimen-specific density threshold settings used in CBCT volumetric analysis introduced operator-dependent variability, and the operator was not formally blinded to group allocation during threshold adjustment. Bur replacement was standardised in this study, with each bur used for a maximum of six specimens and alternated between groups, but some within-bur variation in cutting efficiency could still have occurred across the six permitted uses and cannot be entirely excluded as a contributing factor. Furthermore, tissue losses resulting from attempts to locate canal orifices during conventional access preparation without magnification may occur in any direction, leading to unpredictable root structure destruction and iatrogenic accidents [18]; this pattern is reflected in the greater variability and the presence of the extreme outlier observed in G1. This study did not independently verify that the access cavities achieved were clinically adequate. Reduced volumetric tissue loss should therefore not be equated with a superior clinical outcome without confirmation that access to the root canal system was equivalent between groups; the present findings are restricted to the volumetric endpoint and should not be read as an assessment of access cavity quality. The overall findings should be interpreted as specific to a novice-operator setting and to the imaging protocols used, and do not permit conclusions about the comparative accuracy of CBCT versus IOS in the absence of a reference-standard method such as micro-CT.

From a clinical perspective, these findings demonstrate that magnification reduces volumetric tissue loss during coronal access preparation in mandibular incisors, under the conditions tested. Reduced tissue loss is a biologically plausible mechanistic contributor to reduced iatrogenic events and improved fracture resistance, as reported in the literature [7,10]; however, this remains a hypothesis requiring direct evaluation, since neither access cavity quality nor fracture resistance was assessed in the present study. Where an operating microscope is unavailable, magnification loupes represent a practical alternative. Future research should standardise operator experience and assess whether the volumetric differences demonstrated here are reproduced when operators with equivalent and standardised experience levels perform access preparation in both groups, and should investigate whether these differences translate into measurable outcomes for fracture resistance or long-term tooth survival.

## 5. Conclusions

Within the limitations of this exploratory, single-operator in vitro study, microscope-assisted access cavity preparation resulted in significantly less CBCT-measured volumetric tissue loss than conventional access in mandibular incisors, an effect that remained significant after adjustment for baseline tooth volume and after exclusion of a single outlying specimen. The IOS protocol was restricted to the crown and coronal root third, did not detect the same between-group difference, and showed limited agreement with CBCT for volume lost.

## Figures and Tables

**Table 1:** Descriptive statistics and between-group comparisons for initial, final, and lost dental tissue volumes (mm^3^) measured by cone beam computed tomography according to access cavity preparation method.

CBCT	Conventional Access (G1)	Microscope-Assisted Access (G2)	*p*-Value ^1^	*p*-Value ^2^	95% CI ^3^		
	Mean (SD)	[Min; Max]			Mean (SD)	[Min, Max]	
Initial Volume	250.33 (41.66)	[177.98; 320.80]	230.98 (38.39)	[163.83; 304.55]	0.197	<0.001/<0.001	[−10.61; 49.31]
Final Volume	235.40 (42.84)	[160.97; 306.64]	223.34 (37.79)	[151.91; 294.75]	0.420	<0.001/<0.001	[−18.15; 42.28]
Volume Lost	14.93 (4.42)	[7.80; 26.32]	7.64 (2.72)	[4.27; 11.93]	<0.001	—	[4.54; 10.04]

^1^ Independent-samples Student’s *t*-test (between-group comparison). ^2^ Paired-samples Student’s *t*-test (initial vs. final volume within each group) G1 *p* < 0.001; G2 *p* < 0.001. SD: standard deviation; min: minimum; max: maximum. All values in mm^3^. Cohen’s d for volume lost = 1.99, 95% CI, 1.10–2.88 (large effect). ^3^ 95% CI for the mean difference (G1 – G2).

**Table 2:** Descriptive statistics and between-group comparisons for initial, final, and lost dental tissue volumes measured by intraoral scanning according to access cavity preparation method.

Intraoral Scanner	Conventional Access (G1)	Microscope-Assisted Access (G2)	*p*-Value ^1^	*p*-Value ^2^	95% CI ^3^		
	Mean (SD)	[Min; Max]			Mean (SD)	[Min, Max]	
Initial Volume	215.17 (41.95)	[152.08; 287.99]	201.11 (38.15)	[138.24; 293.70]	0.838	<0.001/<0.001	[−16.66; 50.17]
Final Volume	209.80 (42.60)	[147.17; 278.85]	196.56 (38.40)	[136.84; 292.63]	0.683	<0.001/<0.001	[−16.92; 46.76]
Volume Lost	5.37 (4.17)	[1.17; 14.61]	4.55 (4.08)	[0.86; 12.69]	0.436	—	[−1.80; 2.73]

^1^ Mann–Whitney U test (between-group comparison). ^2^ Wilcoxon signed-rank test (initial vs. final volume within G1/G2, respectively). SD: standard deviation; min: minimum; max: maximum. All values in mm^3^. IOS volumes represent crown and coronal root third only; absolute values are not directly comparable to CBCT values. ^3^ 95% CI for the Hodges–Lehmann shift estimate (G1 − G2), computed from all pairwise between-group differences with confidence limits based on the normal approximation to the Mann–Whitney U distribution.

**Table 3:** Reproducibility analysis between cone beam computed tomography and intraoral scanning using the intraclass correlation coefficient (ICC).

Parameter	ICC	95% CI
Initial Volume	0.732	[−0.034; 0.934]
Final Volume	0.798	[−0.047; 0.951]
Volume Lost	0.011	[−0.150; 0.237]

ICC: intraclass correlation coefficient; CI: confidence interval. ICC values were calculated using a two-way mixed-effects model with absolute agreement [19].

## Data Availability

The individual-specimen volumetric measurements supporting the principal analyses of this study are provided in the Supplementary Materials (Table S1). Additional data are available on request.

## Supplementary Materials

The following supporting information can be downloaded at https://www.mdpi.com/article/10.3390/medicina62081594/s1: Table S1: Individual-specimen volumetric measurements (CBCT and IOS); Figure S1: PRILE 2021 flowchart.

## Abbreviations

The following abbreviations are used in this manuscript:

CBCT	Cone beam computed tomography
IOS	Intraoral scanner
ICC	Intraclass correlation coefficient
SD	Standard deviation
STL	Standard Tessellation Language
DICOM	Digital Imaging and Communications in Medicine
G1	Group 1 (conventional access, without magnification)
G2	Group 2 (microscope-assisted access)
