# Supplementary material for: Evaluating the Role of Magnification in Minimising Tooth Substance Loss During Endodontic Access Cavity Preparation: An In Vitro Volumetric Analysis Using CBCT and IOS
Source: Medicina (Kaunas). 2026 Aug 19;62(8):1594. doi: 10.3390/medicina62081594 (PMC13515486; doi:10.3390/medicina62081594)
Supplement: Supplementary file 1 [file medicina-62-01594-s001.zip › medicina-4465887-supplementary.pdf]

Supplementary Table S1. Individual-specimen volumetric measurements (CBCT and IOS)

Specimen	Group	CBCT			IOS			Note
		Initial Volume	Final Volume	Volume Lost	Initial Volume	Final Volume	Volume Lost	
1	G1 (Conventional)	238.65	223.41	15.25	194.90	192.23	2.67	
2	G2 (Microscope)	304.55	294.75	9.79	293.70	292.63	1.08	
3	G1 (Conventional)	219.34	208.98	10.35	202.52	201.04	1.48	
4	G1 (Conventional)	238.32	220.50	17.82	189.36	186.43	2.93	
5	G1 (Conventional)	303.88	296.08	7.80	277.25	273.57	3.67	
6	G1 (Conventional)	235.84	223.20	12.64	194.90	192.23	2.67	
7	G2 (Microscope)	163.83	151.91	11.93	138.24	136.84	1.40	
8	G1 (Conventional)	247.44	221.12	26.32	212.39	210.00	2.39	
9	G2 (Microscope)	241.06	236.78	4.27	212.89	200.20	12.69	Scanner recorded unusually deep surface capture
10	G2 (Microscope)	187.07	181.56	5.51	159.46	155.55	3.91	
11	G2 (Microscope)	231.72	222.05	9.67	197.85	195.22	2.63	
12	G1 (Conventional)	228.08	218.89	9.19	196.28	192.91	3.37	
13	G2 (Microscope)	233.99	225.28	8.71	191.05	189.86	1.19	
14	G2 (Microscope)	241.12	230.20	10.92	204.27	203.36	0.91	
15	G1 (Conventional)	234.25	216.26	17.99	202.53	187.92	14.61	Scanner recorded unusually deep surface capture
16	G2 (Microscope)	179.55	175.05	4.50	166.09	161.95	4.14	
17	G2 (Microscope)	241.41	235.30	6.11	206.99	200.17	6.81	
18	G1 (Conventional)	212.10	197.33	14.76	176.69	165.80	10.89	
19	G1 (Conventional)	177.98	160.97	17.00	152.08	147.17	4.91	
20	G2 (Microscope)	175.95	171.50	4.45	152.35	147.10	5.25	
21	G1 (Conventional)	297.89	283.77	14.12	265.45	264.28	1.17	
22	G2 (Microscope)	260.15	250.90	9.25	238.91	233.97	4.94	

23	G1 (Conventional)	320.80	306.64	14.17	287.99	278.85	9.13	
24	G1 (Conventional)	273.21	256.22	16.99	220.03	208.71	11.33	
25	G2 (Microscope)	264.77	253.45	11.32	218.58	217.70	0.88	
26	G1 (Conventional)	216.61	200.06	16.54	177.67	171.03	6.64	
27	G1 (Conventional)	310.58	297.64	12.95	277.54	274.87	2.67	
28	G2 (Microscope)	241.27	234.85	6.42	213.23	204.11	9.12	
29	G2 (Microscope)	245.03	238.03	7.00	203.88	191.46	12.41	
30	G2 (Microscope)	253.23	248.53	4.70	219.18	218.32	0.86	

CBCT: cone beam computed tomography; IOS: intraoral scanner. Volume Lost = Initial Volume − Final Volume for the corresponding imaging method. CBCT volumes are converted from the original InVesalius® software output (recorded at 1000× scale) to mm³. IOS volumes represent the crown and coronal root third only (see Methods 2.4); absolute IOS values are therefore not directly comparable to CBCT values for the same specimen. Two specimens (teeth 9 and 15) were annotated in the original data records as instances of unusually deep IOS surface capture. All volumes are reported in mm³.

Figure S1: PRILE 2021 FLOWCHART

RATIONALE: Despite the recognised anatomical complexity of mandibular incisors and the established tissue-conserving benefits of magnification, no prior study had used CBCT-derived volumetric analysis to quantify the effect of microscope magnification on access-cavity tissue loss in this tooth group, or evaluated whether an intraoral scanner (IOS) can reproduce CBCT-derived volumetric estimates for this purpose.

AIM: To compare, using cone beam computed tomography (CBCT) and intraoral scanning (IOS), the volume of dental tissue lost during conventional versus microscope-assisted access cavity preparation in extracted mandibular incisors, and to assess whether IOS can reproduce CBCT-derived volumetric estimates.

ETHICAL APPROVAL: Research ethics committee waiver, Faculty of Dental Medicine, University of Lisbon (Ref. BD-CE-FMDUL202603) – anonymised extracted-teeth-bank specimens exempted under institutional policy.

SAMPLES: Thirty extracted human mandibular incisors (from a pool of 43 screened; 13 excluded for existing restorations, previous endodontic treatment, visible fractures, or extensive caries involving the pulp chamber).

TESTED GROUPS: Group 1 (G1, n=15) – access cavity preparation without magnification. Group 2 (G2, n=15) – access cavity preparation with an operating microscope at ×10 magnification. Allocation by simple random assignment.

OUTCOMES ASSESSED: Volume of dental hard tissue lost during access cavity preparation, determined independently by CBCT and by IOS; inter-method reproducibility (ICC) between CBCT and IOS for initial volume, final volume and volume lost.

METHODS: Pre- and post-preparation CBCT (RAYSCAN® Alpha Plus 130) and IOS (3Shape TRIOS® 3) image acquisition; volumetric segmentation/reconstruction using InVesalius® (CBCT) and Geomagic® Control X (IOS); independent- and paired-samples Student's t-tests (CBCT), Mann–Whitney U and Wilcoxon signed-rank tests (IOS), Cohen's d, two-way mixed-effects intraclass correlation coefficient (ICC), and three post hoc sensitivity analyses (percentage-normalised volume loss, ANCOVA adjusting for initial volume, outlier-exclusion re-analysis).

RESULTS: CBCT analysis revealed significantly greater tissue loss in G1 (mean 14.93 mm^3, SD 4.42) than G2 (mean 7.64 mm^3, SD 2.72; $p<0.001$; Cohen's d=1.99), an effect that persisted after ANCOVA adjustment for initial volume and after exclusion of a single outlying specimen. IOS analysis did not detect a statistically significant between-group difference (5.37 vs. 4.55 mm^3; $p=0.436$). Inter-method reproducibility for volume lost was poor (ICC=0.011, 95% CI −0.150 to 0.237).

CONCLUSIONS: Within the limitations of this exploratory, single-operator in vitro study, microscope-assisted access cavity preparation resulted in significantly less CBCT-measured volumetric tissue loss than conventional access in mandibular incisors. The IOS protocol, restricted to the crown and coronal root third, did not detect the same between-group difference and showed poor agreement with CBCT for volume lost.

FUNDING: This research received no external funding.

CONFLICTS: The authors declare no conflicts of interest.

***From: Nagendrababu V, Murray PE, Ordinola-Zapata R, Peters OA, Rôças IN, Siqueira JF Jr, Priya E, Jayaraman J, Pulikkotil SJ, Camilleri J, Boutsioukis C, Rossi-Fedele G, Dummer PMH (2021) PRILE 2021 guidelines for reporting laboratory studies in Endodontology: a consensus-based development. *International Endodontic Journal* May 3. doi: 10.1111/iej.13542. https://onlinelibrary.wiley.com/doi/abs/10.1111/iej.13542.**

For further details visit: http://pride-endodonticguidelines.org/prile
